# Admission of patients with chest pain and/or breathlessness from the emergency department in relation to risk assessment and copeptin levels – an observational study

**DOI:** 10.48101/ujms.127.8941

**Published:** 2022-12-26

**Authors:** Lee Ti Davidson, Emilia Gauffin, Preben Henanger, Maciej Wajda, Daniel Wilhelms, Bertil Ekman, Hans J. Arnqvist, Martin Schilling, Simona I. Chisalita

**Affiliations:** a Department of Emergency Medicine in Linköping, Local Health Care Services in Central Östergötland, Region Östergötland & Department of Biomedical and Clinical Sciences, Linköping University, Sweden; b Department of Endocrinology in Linköping, and Department of Health, Medicine and Caring Sciences, Linköping University, Linköping, Sweden; c Clinicum and Innovations Centrum, Department of Emergency Medicine and Department of Clinical and Experimental Medicine, Linköping University, Linköping, Sweden

**Keywords:** Emergency department, chest pain, breathlessness, copeptin, MR-proADM, MR-proANP

## Abstract

**Background:**

One of the most critical decisions that emergency department (ED) physicians make is the discharge versus admission of patients. We aimed to study the association of the decision in the ED to admit patients with chest pain and/or breathlessness to a ward with risk assessment using the Rapid Emergency Triage and Treatment System (RETTS), the National Early Warning Score (NEWS), and plasma levels of the biomarkers copeptin, midregional proadrenomedulin (MR-proADM), and midregional proatrial natriuretic peptide (MR-proANP).

**Methods:**

Patients presenting at the ED with chest pain and/or breathlessness with less than one week onset were enrolled. Patients were triaged according to RETTS. NEWS was calculated from the vital signs retrospectively.

**Results:**

Three hundred and thirty-four patients (167 males), mean age 63.8 ± 16.8 years, were included. Of which, 210 (62.8%) patients complained of chest pain, 65 (19.5%) of breathlessness, and 59 (17.7%) of both. Of these, 176 (52.7%) patients were admitted to a ward, and 158 (47.3%) patients were discharged from the ED. In binary logistic models, age, gender, vital signs (O_2_ saturation and heart rate), NEWS class, and copeptin were associated with admission to a ward from the ED. In receiver-operating-characteristics (ROC) analysis, copeptin had an incremental predictive value compared to NEWS alone (*P* = 0.002).

**Conclusions:**

Emergency physicians’ decisions to admit patients with chest pain and/or breathlessness from the ED to a ward are related to age, O_2_ saturation, heart rate, NEWS category, and copeptin. As an independent predictive marker for admission, early analysis of copeptin might be beneficial when improving patient pathways at the ED.

## Background

Chest pain and/or breathlessness are common symptoms in patients attending the emergency department (ED), and both have a broad spectrum of underlying diseases (1, 2). A critical issue for the emergency physician at the ED is to decide which patients should be admitted to a ward and which patients can be discharged home (1, 3–5).

The Rapid Emergency Triage Treatment Scale (RETTS©) (6) is used to identify and prioritize patients who need acute treatment and to reduce waiting time at the ED 6, 7). Based on their presenting symptoms and vital signs, patients are allocated to five triage priority categories (blue, green, yellow, orange, and red), based on cut-off levels for vital signs and chief complaint algorithms, resulting in different recommended times for physician assessment (6).

The National Early Warning Score (NEWS) is a track and trigger system developed on hospital wards by assessing patients’ vital signs in order to detect patients at high risk of serious adverse events, such as unplanned intensive care unit (ICU) admission, cardiac arrest, or in-hospital death within 24 h (8–14). NEWS has been validated in EDs and in hospital settings (11, 12, 15). Triage scores characterize a patient’s clinical state at a single time point. When vital signs are still unremarkable, patients at risk of deterioration might be missed.

Copeptin, MR-proADM, and MR-proANP are surrogate markers for arginine vasopressin (AVP), adrenomedullin (ADM), and atrial natriuretic peptide (ANP), respectively (16, 17). These biomarkers have shown to improve diagnostic performance and risk stratification of patients presenting with chest pain and/or breathlessness at the ED (18–20). AVP is secreted from the posterior pituitary gland and mediates vasoconstriction and water retention. In an ED, adding copeptin analysis to troponin improves the diagnostic reliability of acute coronary syndrome compared to troponin alone (21). Stretching of myocytes leads to the secretion of ANP from the heart’s atria, which promotes natriuresis, diuresis, vasodilation, and inhibition of the renin angiotensin aldosterone system (22). MR-proANP has similar diagnostic and prognostic performance to other natriuretic peptides in heart failure (23, 24). ADM is secreted from the endothelial cells in the vessel wall as a result of ischemia and is, hence, associated with endothelial dysfunction (18). Higher levels of MR-proADM are associated with adverse outcomes in patients with cardiovascular diseases (23–25).

It is vital that the physician can identify the patients who need to be admitted to a ward, so these patients can achieve the best possible care. Moreover, fast assessment of patients without need of in-hospital care shortens the waiting time at the ED, which has been shown to correlate with adverse outcomes (26). Risk assessment tools such as RETTS and NEWS are used at ED to prioritize and assess the patients. Our hypothesis is that biomarkers such as copeptin, MR-proADM, and MR-proANP could aid emergency physicians by identifying patients in need of admission. This study aimed to investigate the association of vital signs and risk assessment using RETTS, NEWS, and biomarkers with the decision on admission to a ward from the ED in patients with chest pain and/or breathlessness.

## Methods

### Study design

We conducted a single-center observational study in the ED at a tertiary care teaching hospital with a primary catchment area of 460,000 inhabitants and 48,000 ED visits annually during the period 2013–2017.

Non-critically ill patients 18 years or older presenting at the ED with chest pain, breathlessness, or both, with the onset within 7 days, were enrolled in this study after providing a written informed consent. Exclusion criteria included advanced renal failure, advanced malignancy, liver failure, ST-segment elevation myocardial infarction or new left bundle branch block, and severely ill patients (pronounced shortness of breath, massive chest pain, or clinically unstable patients for whom there was no doubt about requiring urgent medical attention).

Blood pressure, respiratory rate, heart rate, body temperature, and oxygen saturation evaluated by pulse oximetry were recorded. Patients were triaged by a nurse according to the Rapid Emergency Triage and Treatment System (RETTS) (Figure 1 in the supplementary material). The triage priorities (in order of acuity) are red, orange, yellow, green, and blue, with red being the highest priority and blue being the lowest. The patients were dichotomized as either low (blue, green, or yellow) or high (red or orange) priority in the statistical analyses.

The NEWS was retrospectively calculated using the recorded vital signs, i.e. respiratory rate, oxygen saturation, temperature, blood pressure, pulse rate, and level of consciousness, as described previously by the Royal College of Physicians (13). Using the resulting combined score, patients were classified into three NEWS categories representing low (0–4 points), moderate (5–6 points), and high (≥7 points) risk (Figure 2a and 2b in the supplementary materials). Because data on supplemental oxygen were not available, the results presented here correspond to NEWS-potentially minus 2, i.e. adjusted NEWS.

All patient management was performed by clinical routine.

### Biochemical analyses

Blood samples were collected on presentation at the ED. Plasma was separated by the core laboratory and kept frozen at −70°C for later analysis. Copeptin, MR-proADM, and MR-proANP were analyzed using a highly sensitive time-resolved amplified cryptate emission technology assay (B·R·A·H·M·S, KRYPTOR, AG, *Hennigsdorf*, Germany). The assay has a lower detection limit of 1.7 pmol/L and an inter-assay precision of 5.2% CV for copeptin. For MR-proADM, the assay has an analytical detection limit of 0.04 nmol/L and an inter-assay variability of 3.3%. For MR-proANP, the assay has a detection limit of 2.1 pmol/L and an inter-assay variability of 3.0%.

Blood samples for C-reactive protein (CRP), creatinine, and high sensitivity Troponin T (hsTNT) were analyzed at the central laboratory by clinical standard.

All clinical information including the final diagnosis was collected from Cambio COSMIC^®^ digital medical records by two physicians (S.I.C. and L.T.D.), with a follow-up of 90 days after the initial presentation.

### Main outcome

The outcome of this study was admission to a ward from the ED, coded as a binary variable: admitted to a ward or discharged from the ED.

### Ethics

All participants gave their written informed consent. This study was approved by the Regional Ethical Review Board in Linköping, Sweden (diary number 2011/501-31). The study protocol followed the principles expressed in the Declaration of Helsinki.

### Statistical analysis

The statistical analyses were performed using IBM SPSS Statistics V.28.0.1.0. Means and standard deviation, median and interquartile ranges, counts, and percentages were reported as appropriate. The mean and median between groups were compared using either t-test or median test for continuous numerical data and Chi-square test for categorical variables. *P*-values < 0.05 were considered statistically significant. The biomarkers MR-proANP, MR-proADM, and copeptin were dichotomized by cut-off values used in previous studies (23, 27, 28). Data were analyzed with binary logistic regression to estimate adjusted odds ratio (ORs) for the single outcome variable identified above. First, univariable analysis was performed for age, gender, vital signs, NEWS, RETTS, copeptin, MR-proADM, or MR-proANP. Thereafter, three different models of multivariable analysis were performed: (1) age, gender, vital signs, copeptin, MR-proADM, and MR-proANP; (2) age, gender, NEWS moderate/high versus low class, copeptin, MR-proADM, and MR-proANP; and (3) age, gender, RETTS high versus low priority, copeptin, MR-proADM, and MR-proANP. Finally, a supplemental analysis was performed adjusting the previous models for creatinine and hsTNT.

Receiver-operating-characteristics (ROC) curves were constructed for NEWS separately and for NEWS combined with continuous copeptin to determine the prognostic performance with area-under-the-curves (AUCs) for prediction of admission to a ward. We used Stata (MP v17.1, College Station, USA) to test for differences between the AUCs.

## Results

In total, 334 patients with a mean age of 63.8 ± 16.8 years were included in the study. Baseline data for the whole population and the admitted and discharged patients are presented in Table 1. Of all patients, 210 complained of chest pain (62.8%), 65 (19.5%) of breathlessness, and 59 (17.7%) of chest pain and breathlessness.

**Table 1:** Baseline characteristics of the study population.

Variables	All population	Discharged from ED	Admitted to a ward	*P*-value
Number of patients	334	158	176	
Age, years (mean ± SD)	63.79 ± 16.87	57.26 ± 18.30	69.66 ± 12.97	< 0.001
Male/female, *n* (%)	167 (50.0)/167 (50.0)	68 (43.0)/90 (57.0)	99 (56.3)/77 (43.8)	0.016
Chest pain, *n* (%)	210 (62.8)	105 (66.4)	105 (59.6)	0.119
Breathlessness, *n* (%)	65 (19.5)	32 (20.3)	33 (18.8)	0.131
Chest pain and breathlessness, *n* (%)	59 (17.7)	21 (13.3)	38 (21.6)	0.047
NEWS low, *n* (%)	283 (90.1)	144 (97.3)	139 (83.7)	< 0.001
NEWS moderate, *n* (%)	16 (5.1)	3 (2.0)	13 (7.8)	0.020
NEWS high, *n* (%)	15 (4.8)	1 (0.7)	14 (8.4)	0.001
RETTS low (yellow, green, and blue), *n* (%)	97 (34.9)	52 (42.6)	45 (28.8)	0.017
RETTS orange, *n* (%)	170 (61.2)	69 (56.6)	101 (64.7)	0.165
RETTS red, *n* (%)	11 (4.0)	1 (0.8)	10 (6.4)	0.018
RETTS high (red + orange), *n* (%)	181 (65.1)	70 (57.4)	111 (71.2)	0.017
Blood pressure systolic, mmHg (mean ± SD)	147.36 ± 23.79	146.43 ± 21.98	148.20 ± 25.33	0.49
Blood pressure diastolic, mmHg (mean ± SD)	83.32 ± 14.35	84.05 ± 13.62	82.68 ± 14.98	0.39
Temperature, °C (mean ± SD)	36.93 ± 0.68	37.00 ± 0.60	36.86 ± 0.74	0.79
Saturation, % (mean ± SD)	96.46 ± 3.88	97.75 ± 2.36	95.30 ± 4.57	< 0.001
Respiratory rate/minute (mean ± SD)	18.95 ± 5.21	18.32 ± 4.79	19.51 ± 5.52	0.04
Heart rate/minute (mean ± SD)	80.34 ± 20.14	75.76 ± 15.40	84.45 ± 22.88	< 0.001
Length of hospital stay, days (median (IQR))	1 (0–3)	0 (0.0)	2 (1–5)	< 0.001
Previous ischemic heart disease	89 (26.6)	26 (16.5)	63 (35.8)	< 0.001
Comorbidities	240 (72.1)	94 (59.9)	146 (83.0)	< 0.001
Copeptin, nmol/L (median (IQR))	6.20 (3.69–13.49)	4.94 (3.34–8.11)	9.58 (4.05–26.58)	< 0.001
MR-proADM, nmol/L (median (IQR))	0.68 (0.53–0.90)	0.61 (0.33–0.47)	0.73 (0.58–1.06)	< 0.001
MR-proANP, pmol/L (median (IQR))	91.05 (57.29–179.43)	72.75 (47.04–115.10)	126.75 (73.33–244.08)	< 0.001
Creatinine, µmol/L (median (IQR))	79 (65–97)	75 (65–94)	81 (66.0–102)	0.004
High sensitivity troponin T, µmol/L (median (IQR))	9 (6–19)	7 (5–10)	13 (7–27)	0.246

Missing values: NEWS 20, RETTS 56, systolic BP 1, diastolic BP 9, temperature 11, saturation 1, respiratory rate 10, copeptin 20, MR-proADM 25, and MR-proANP 25.

After examination at the ED, 176 (52.7%) patients were admitted, and 158 (47.3%) patients were discharged. The admitted patients had a hospitalization time of 1 (0; 3) day (median; interquartile range (IQR)). No patients were admitted to the ICU. Patients admitted to a ward tended to be older and predominately male compared with those who were discharged from the ED. There was a significant difference in oxygen saturation (O_2_ saturation), respiratory rate, and heart rate between admitted and discharged patients (Table 1).

In all, 89 (26.6%) had at least one prior episode of ischemic heart disease (e.g. unstable angina, myocardial infarction, coronary artery bypass grafting, or percutaneous coronary intervention), whereas most of the patients (240, 72.1%) had at least one comorbidity (e.g. hypertension, ischemic heart disease, atrial fibrillation, heart failure, stroke, transient ischemic attack, thromboembolism, diabetes mellitus, renal dysfunction, asthma, chronic obstructive pulmonary disease, systemic inflammatory diseases, or malignancy).

The final diagnoses are presented in Table 2. In total, 68 patients were admitted to a ward for observation and thereafter discharged with a non-specific diagnosis. Of these, 51 (75%) had a copeptin value below 10 nmol/L.

**Table 2:** Diagnosis at discharge from the hospital in patients who were admitted to a ward.

Diagnosis	Copeptin ≤ 10 nmol/L	Copeptin >10 nmol/L	Total (*n*)
Non-specific diagnosis	51 (75.0%)	17 (25.0%)	68
Acute coronary syndrome	13 (43.3%)	17 (56.7%)	30
Other cardiac diagnosis	16 (35.6%)	29 (64.4%)	45
Acute lung infection	2 (15.4%)	11 (84.6%)	13

Using the RETTS triage model, 97 (34.9%) of the patients were classified as low priority (blue, green, or yellow) and 181 (65.1%) as high priority (red or orange) (Table 1). In the group assigned RETTS high priority, the number of patients admitted to a ward was higher than those discharged home (111 [71.2%] vs. 70 [28.8%], *P* = 0.017).

In the NEWS track and trigger system model, most patients assigned to the moderate (13 [7.8%] vs. 3 [2.0%], *P* = 0.02) and high (14 [8.4%] vs. 1 [0.7%], *P* = 0.001) risk groups were admitted. Levels of copeptin, MR-proADM, and MR-proANP were significantly higher in the patients admitted to a ward (Table 1). In Table 3, the levels of the biomarkers are presented according to group of RETTS and NEWS.

**Table 3:** Median (IQR) levels of copeptin, MR-proADM, and MR-proANP across the categories of NEWS and RETTS.

Biomarkers	RETTS low	RETTS high	*P*-value	NEWS low	NEWS moderate + high	*P*-value
Copeptin (nmol/L)	4.94 (3.59–11.67)	8.56 (4.03–22.10)	0.030	5.71 (3.48–11.54)	27.92 (10.48–56.26)	<0.001
MR-proADM (pmol/L)	0.63 (0.50–0.83)	0.70 (0.57–1.07)	0.360	0.65 (0.52–0.88)	1.07 (0.78–1.56)	0.003
MR-proANP (nmol/L)	89.49 (47.61–174.70)	106.80 (65.33–236.50)	0.035	88.40 (55.54–163.10)	204.80 (70.93–354.60)	0.006

None of the discharged patients died within 90 days of follow-up, while there were five deaths in the group admitted to a ward.

The association of vital signs, NEWS, and biomarkers with admission to a ward or discharge from the ED was tested in multivariate binary regression models (Tables 4a-c). In model 4a, age (OR 1.031 [1.006–1.057]), O_2_ saturation (OR 0.860 [0.775–0.954]), heart rate (OR 1.020 [1.003–1.037]), and copeptin >10 nmol/L (OR 2.254 [1.116–4.549]) were associated with admission to a ward. When the compound scale NEWS was used instead of single vital signs, an association with admission to a ward was found (OR 3.592 [1.082–11.991]) (Table 4b), while no significant association between RETTS and the need for admission could be found (*P* = 0.204) (Table 4c). Even in these models, copeptin remained significantly associated with the outcome of admission/discharge (OR 2.308 [1.163–4.582] and OR 2.644 [1.272–5.497]) (Table 4b-c). After adjusting model 4a–c for creatinine and hsTNT (supplementary materials table 1), only copeptin maintained the significance in all models (OR 2.662 [1.064–6.657], OR 2.795 [1.150–6.792], and OR 3.792 [1.444–9.954], respectively), whereas age, saturation, heart rate, and NEWS lost their predictive value for admission to a ward.

**Table 4:** Binary regression analyses for associations of age, gender, vital signs (a), NEWS (b), RETTS (c), and biomarkers with admission to a ward.

Variables in the predicting model	Univariable		Multivariable	
	OR (95%CI)	*P*-value	OR (95%CI)	*P*-value
Age	1.051 (1.035–1.067)	< 0.001	1.031 (1.006–1.057)	0.014
Gender	1.702 (1.103–2.625)	0.016	1.365 (0.763–2.444)	0.295
Systolic blood pressure	1.003 (0.994–1.012)	0.497	0.998 (0.986–1.010)	0.734
Temperature	0.746 (0.537–1.037)	0.081	0.647 (0.403–1.040)	0.072
Saturation	0.781 (0.712–0.856)	< 0.001	0.860 (0.775–0.954)	0.004
Respiratory rate	1.049 (1.001–1.100)	0.045	0.985 (0.918–1.056)	0.667
Heart rate	1.023 (1.011–1.035)	< 0.001	1.020 (1.003–1.037)	0.021
Copeptin > 10 nmol/L	4.569 (2.668–7.826)	< 0.001	2.254 (1.116–4.549)	0.023
MR-proADM > 0.75 pmol/L	2.500 (1.553–4.023)	< 0.001	0.797 (0.382–1.664)	0.546
MR-proANP > 120 nmol/L	3.513 (2.149–5.741)	< 0.001	1.369 (0.642–2.919)	0.416


b.	Univariable OR (95%CI)	*P*-value	Multivariable OR (95%CI)	*P*-value

Age	1.051 (1.035–1.067)	< 0.001	1.034 (1.011–1.058)	0.004
Gender	1.702 (1.103–2.625)	0.016	1.665 (0.953–2.909)	0.073
NEWS high + moderate vs. low	6.993 (2.385–20.502)	< 0.001	3.592 (1.082–11.991)	0.037
Copeptin > 10 nmol/L	4.569 (2.698–11.416)	< 0.001	2.308 (1.163–4.582)	0.017
MR-proADM > 0.75 pmol/L	2.500 (1.553–4.023)	0.005	0.774 (0.386–1.551)	0.470
MR-proANP > 120 nmol/L	3.513 (2.149–5.741)	< 0.001	1.689 (0.814–3.507)	0.160


c.	Univariable OR (95%CI)	*P*-value	Multivariable OR (95%CI)	*P*-value

Age	1.051 (1.035–1.067)	< 0.001	1.038 (1.013–1.063)	0.003
Gender	1.702 (1.103–2.625)	0.016	1.906 (1.055–3.442)	0.033
RETTS high vs. low	1.832 (1.113–3.017)	0.017	1.464 (0.813–2.637)	0.204
Copeptin > 10 nmol/L	4.569 (2.668–7.826)	< 0.001	2.644 (1.272–5.497)	0.009
MR-proADM > 0.75 pmol/L	2.500 (1.553–4.023)	0.005	0.467 (0.212–1.030)	0.059
MR-proANP > 120 nmol/L	3.513 (2.149–5.741)	< 0.001	1.886 (0.858–4.145)	0.114

Notes: ED: emergency department; BP: blood pressure; MR-proADM: midregional proadrenomedulin; MR-proANP: midregional proatrial natriuretic peptide; CRP: C-reactive protein; RETTS: Rapid Emergency Triage and Treatment System; NEWS: National Early Warning Score; OR: odds ratio; CI: confidence interval.

**Figure 1:**
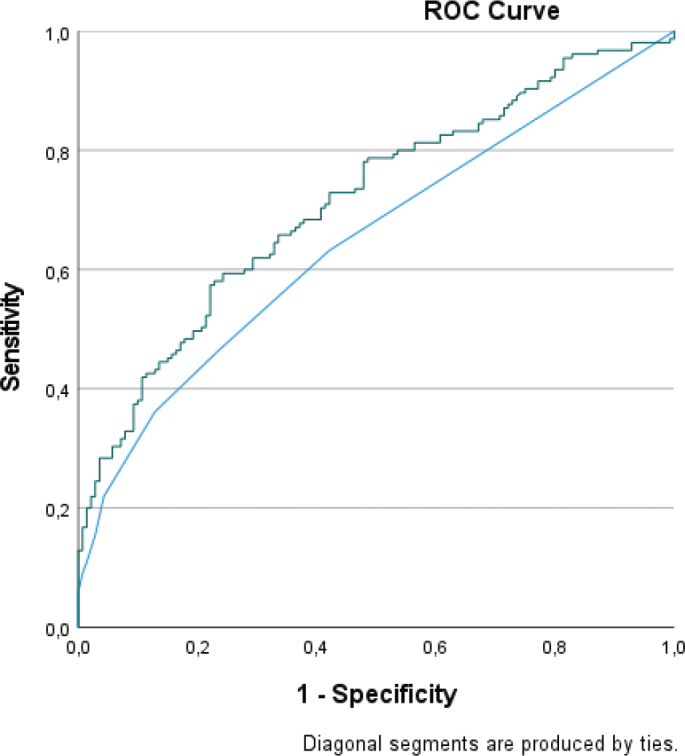
ROC curve for prediction of admission to a ward. Blue = NEWS. Green = NEWS + copeptin.

In ROC analysis, NEWS showed an AUC of 0.649 (0.591–0.707) and the combination of NEWS and copeptin showed an AUC of 0.711 (0.652–0.769), see Figure 1. Adding copeptin had a significant incremental predictive value when compared to analysis of NEWS separately (*P* = 0.002).

## Discussion

In this study, we found that age, gender, vital signs, NEWS, and copeptin were associated with the decision of admission to a ward for patients presenting at the ED with chest pain and/or breathlessness. Vital signs such as heart rate, respiratory rate, O_2_ saturation, temperature, blood pressure, and consciousness objectively indicate the immediate well-being of patients and are an imperative component of patient assessment and management (29). In our study, O_2_ saturation, heart rate, and age were independently associated with the decision on admission to a ward. In geriatric patients, knowledge of pulse oximetry values has been shown to affect the decision on admission (30). Heart rate on admission is associated with prognosis in patients with heart failure (31). In a large, unselected ED population, age and vital signs were significantly related to 1-day mortality, 30-day mortality, and ICU admission (32). In the same study, respiratory rate and oxygen saturation were associated with higher odds of mortality than changes in systolic blood pressure and pulse rate.

The patients in our study were risk-stratified according to the RETTS priority triage model, which is used to identify and prioritize the order in which patients need to be dealt with by the ED physician (6, 7). In our study, the proportion of patients classified in the red RETTS priority class was 4.0%, which is similar to results presented by Ljunggren et al. (32). By contrast, the proportion of patients allocated to the orange RETTS priority class, 61.2%, was considerably higher than the 6.7% reported by the same authors (32). It is conceivable that in our study, there might have been overtriage due to the broad triage criteria in RETTS. It has to be noted that in RETTS, a higher triage level can be applied by triage nurses’ discretion. Ljunggren et al. suggested that in RETTS, the most commonly used triage system in Sweden, future triage systems should also include age (32).

With NEWS, a lower number of patients were included in the high and moderate risk classes: 4.8% and 5.1% vs. 90.1% in the low NEWS risk class. It should be pointed out that the emergency physicians were aware of vital signs and the RETTS priority classes, but not specifically of the NEWS score and were blinded towards the biomarkers studied.

In the multivariate regression model including age and sex, there was no significant difference between high (red and orange) and low (green, blue, and yellow) RETTS levels regarding admission to a ward, whereas a significant difference was found for NEWS risk levels between patients admitted to a ward and those discharged home. This suggests that the track and trigger system NEWS is a more sensitive tool for risk stratification than triage priority using the RETTS model. Few studies have previously considered the predictive value of NEWS for admission to a ward (9, 33). Mitsunaga and colleagues found that NEWS values in the ED effectively predicted admission to a ward and in-hospital mortality in elderly patients (9). Other studies have investigated the discriminative power of NEWS in the ED for admission to an ICU ward and for all-cause mortality (10, 15, 34, 35). Recent studies have shown that the Swedish version of NEWS had excellent inter-rater reliability, and the median scores for patients admitted to the ICU were higher than for those not admitted. Patients classified as medium or high risk by NEWS experienced a twofold or threefold increase, respectively, in odds of in-hospital death or 30-day mortality compared to low-risk patients (11, 12).

In our study, copeptin was independently correlated to admission to a ward in addition to age, gender, vital signs, and NEWS, whereas MR-proADM and MR-proANP were not. This suggests that copeptin could be used at the ED to aid emergency physician evaluating the need of admission. In a study of patients presenting with non-specific complaints, elevated copeptin was associated with increased 30-day mortality (36). In a large, multicenter, unselected ED cohort of patients, Schuetz and colleagues showed that combining clinical information and measured copeptin, MR-proADM and procalcitonin strongly predicted high initial triage priority, ICU admission, and 30-day mortality (37). The clinical examination is fundamental, and the patients will still need to be prioritized, for example, by vital parameters or NEWS, but levels of copeptin could give additional aid in the decision-making. In our study, the ROC-curve for the model including copeptin showed a significant incremental predictive value for admission to the use of NEWS solely.

In our study, 68 patients were discharged from a ward with a non-specific diagnosis. Many patients have probably unnecessarily been admitted to a ward due to lack of reliable risk stratification methods. Analysis of copeptin might have helped the physician to discharge some of these patients directly from the ED. Previous studies have shown that ED physicians receive little training on how to make disposition decisions, and that the applied research in this area has focused on the implementation of decision rules or algorithms for narrow, predefined patient groups, such as those presenting with chest pain (38–40). Cardel et al. have studied in ‘real time’ how experienced ED physicians make discharge decisions and reported that they most often relied on clinical judgment, rather than evidence-based guidelines (5). This aspect could also have implications for the results of our study.

A strength of our study is the broad inclusion criteria consistent with daily practice. We believe that the unselected feature of the cohort makes it representative of patients typically seen in clinical practice in the ED with a well-distributed gender balance, i.e. the same number of male and female participants. However, the sample size is small compared to the number of patients presenting with chest pain and/or breathlessness each year at the ED.

Further study limitations include the fact that the decision on admission to a ward was taken by several ED physicians with different levels of experience and may, therefore, be subject to variation. Also, physicians could not be blinded to the triage score, which might affect initial clinical management due to priority recommendations connected to triage. Finally, information on the use of oxygen was not available for the retrospective calculation of NEWS, reducing the maximum score to 18 out of 20. Therefore, in accordance with the Royal College of Physicians, a weighting score of two was added. This could result in the misclassification of NEWS categories. Furthermore, the use of hsTNT as diagnostic biomarker in the clinical pathway for chest pain patients directly related to the decision to admit might have outweighed the potential benefit of NEWS in our study with the majority of patients (90.1%) presenting with low NEWS scores.

## Conclusions

In conclusion, emergency physicians’ decisions to admit patients with chest pain and/or breathlessness from the ED to a ward are related to age, O_2_ saturation, heart rate, NEWS category, and copeptin. As an independent predictive marker for admission, early analysis of copeptin might be beneficial when improving patient pathways at the ED. Further prospective studies evaluating the value of copeptin on top of clinical judgement and NEWS seem motivated.

## Supplementary Material

Admission of patients with chest pain and/or breathlessness from the emergency department in relation to risk assessment and copeptin levels – an observational study
