# Supplementary material for: Admission of patients with chest pain and/or breathlessness from the emergency department in relation to risk assessment and copeptin levels – an observational study
Source: Ups J Med Sci. 2022 Dec 26;127:10.48101/ujms.127.8941. doi: 10.48101/ujms.127.8941 (PMC9793763; doi:10.48101/ujms.127.8941)
Supplement: Admission of patients with chest pain and/or breathlessness from the emergency department in relation to risk assessment and copeptin levels – an observational study [file UJMS-127-8941-s001.pdf]

Supplementary Materials:

Figure 1.

Cut-off levels due to vital signs (VS), according to RETTS©, example*: Chest pain, algorithm no. 5,* free translation after description in Widgren (6).

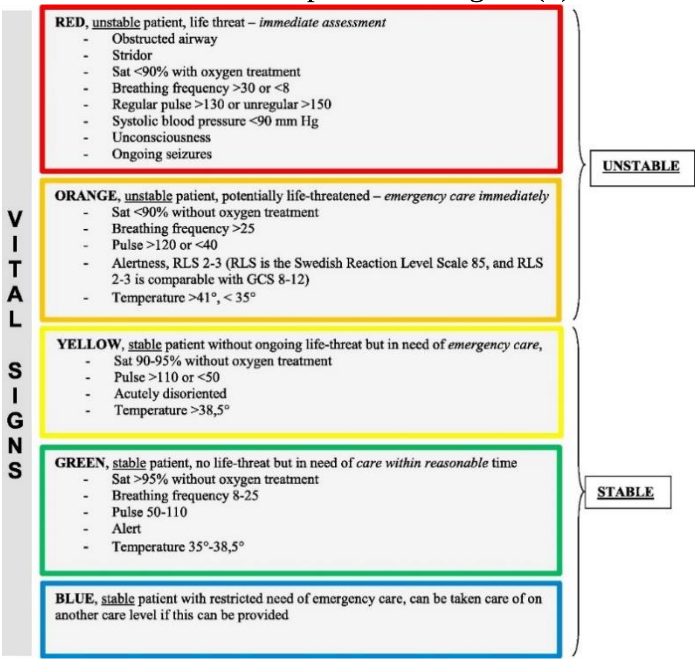

Figure 2a. The NEWS scoring system (14).

Physiological parameter	Score 3	2	1	0	1	2	3
Respiration rate (per minute)	≤8		9–11	12–20		21–24	≥25
SpO_2 Scale 1 (%)	≤91	92–93	94–95	≥96			
SpO_2 Scale 2 (%)	≤83	84–85	86–87	88–92<br>≥93 on air	93–94 on oxygen	95–96 on oxygen	≥97 on oxygen
Air or oxygen?		Oxygen		Air			
Systolic blood pressure (mmHg)	≤90	91–100	101–110	111–219			≥220
Pulse (per minute)	≤40		41–50	51–90	91–110	111–130	≥131
Consciousness				Alert			CVPU
Temperature (°C)	≤35.0		35.1–36.0	36.1–38.0	38.1–39.0	≥39.1	

Figure 2b. NEWS thresholds and triggers (14).

NEW score	Clinical risk	Response
Aggregate score 0–4	Low	Ward-based response
Red score<br>Score of 3 in any individual parameter	Low–medium	Urgent ward-based response*
Aggregate score 5–6	Medium	Key threshold for urgent response*
Aggregate score 7 or more	High	Urgent or emergency response**

* Response by a clinician or team with competence in the assessment and treatment of acutely ill patients and in recognising when the escalation of care to a critical care team is appropriate.
**The response team must also include staff with critical care skills, including airway management.

NEW score	Frequency of monitoring	Clinical response
0	Minimum 12 hourly	• Continue routine NEWS monitoring
Total 1–4	Minimum 4–6 hourly	• Inform registered nurse, who must assess the patient<br>• Registered nurse decides whether increased frequency of monitoring and/or escalation of care is required
3 in single parameter	Minimum 1 hourly	• Registered nurse to inform medical team caring for the patient, who will review and decide whether escalation of care is necessary
Total 5 or more Urgent response threshold	Minimum 1 hourly	• Registered nurse to immediately inform the medical team caring for the patient<br>• Registered nurse to request urgent assessment by a clinician or team with core competencies in the care of acutely ill patients<br>• Provide clinical care in an environment with monitoring facilities
Total 7 or more Emergency response threshold	Continuous monitoring of vital signs	• Registered nurse to immediately inform the medical team caring for the patient – this should be at least at specialist registrar level<br>• Emergency assessment by a team with critical care competencies, including practitioner(s) with advanced airway management skills<br>• Consider transfer of care to a level 2 or 3 clinical care facility, ie higher-dependency unit or ICU<br>• Clinical care in an environment with monitoring facilities

Table 1. Binary regression analyses for associations of age, gender, vital signs, NEWS, RETTS and biomarkers with admission to a ward.

**a.**	**Univariable**		**Multivariable**	
	**OR (95%CI)**	**p-value**	**OR (95%CI)**	**p-value**
Age	1.051 (1.035-1.067)	<0.001	1,.023 (0.991-1.057)	0.161
Male	1.702 (1.103-2.625)	0.016	1.885 (0.842-4.217)	0.123
Systolic blood pressure	1.003 (0.994-1.012)	0.497	1.001 (0.986-1.016)	0.873
Temperature	0.746 (0.537-1.037)	0.081	0.525 (0.274-1.006)	0.052
Saturation	0.781 (0.712-0.856)	<0.001	0.888 (0.780-1.012)	0.074
Respiratory rate	1.049 (1.001-1.100)	0.045	1.049 (0.951-1.156)	0.339
Heart rate	1.023 (1.011-1.035)	<0.001	1.015 (0.995-1.036)	0.152
Creatinine	1.013 (1.003-1.024)	0.009	1.000 (0.982-1.019)	0.997
Troponin T	1.051 (1.025-1.077)	<0.001	1.013 (0.991-1.035)	0.256
Copeptin>10 nmol/L	4.569 (2.668-7.826)	<0.001	2.662 (1.064-6.657)	0.036
MR-proADM>0.75 pmol/L	2.500 (1.553-4.023)	<0.001	0.770 (0.272-2.179)	0.623
MR-proANP>120 nmol/L	3.513 (2.149-5.741)	<0.001	0.977 (0.366-2.607)	0.964

**b.**	**Univariable**		**Multivariable**	
	**OR (95%CI)**	**p-value**	**OR (95%CI)**	**p-value**
Age	1.051 (1.035-1.067)	<0.001	1.025 (0.994-1.057)	0.109
Male	1.702 (1.103-2.625)	0.016	1.801 (0.829-3.914)	0.137
NEWS high+moderate vs. low	6.993 (2.385-20.502)	<0.001	1.754 (0.415-7.413)	0.445
Creatinine	1.013 (1.003-1.024)	0.009	0.999 (0.981-1.017)	0.881
Troponin T	1.051 (1.025-1.077)	<0.001	1.016 (0.988-1.045)	0.262
Copeptin>10 nmol/L	4.569 (2.698-11.416)	<0.001	2.795 (1.150-6.792)	0.023
MR-proADM>0.75 pmol/L	2.500 (1.553-4.023)	0.005	0.752 (0.286-1.975)	0.563
MR-proANP>120 nmol/L	3.513 (2.149-5.741)	<0.001	1.321 (0.517-3.375)	0.560

c.	**Univariable OR (95%CI)**	**p-value**	**Multivariable OR (95%CI)**	**p-value**
Age	1.051 (1.035-1.067)	<0.001	1.034 (1.001-1.068)	0.045
Male	1.702 (1.103-2.625)	0.016	2.362 (1.053-5.296)	0.037
RETTS high vs. low	1.832 (1.113-3.017)	0.017	0.971 (0.459-2.051)	0.938
Creatinine	1.013 (1.003-1.024)	0.009	0.998 (0.981-1.015)	0.799
Troponin T	1.051 (1.025-1.077)	<0.001	1.013 (0.984-1.043)	0.385
Copeptin>10 nmol/L	4.569 (2.668-7.826)	<0.001	3.792 (1.444-9.954)	0.007
MR-proADM>0.75 pmol/L	2.500 (1.553-4.023)	0.005	0.358 (0.122-1.052)	0.062
MR-proANP>120 nmol/L	3.513 (2.149-5.741)	<0.001	1.572 (0.588-4.201)	0.367

Notes: ED = emergency epartment, BP = blood pressure, MR-proADM = midregional proadrenomedulin, MR-proANP = midregional proatrial natriuretic peptide, CRP = C-reactive protein, RETTS = Rapid Emergency Triage and Treatment System, NEWS = National Early Warning Score, OR = odds ratio, CI = confidence interval.
